# Protective effect of *Panax quinquefolium* 20(S)-protopanaxadiol saponins, isolated from *Pana quinquefolium*, on permanent focal cerebral ischemic injury in rats

**DOI:** 10.3892/etm.2013.1405

**Published:** 2013-11-12

**Authors:** HUALI XU, XIAOFENG YU, SHAOCHUN QU, YANGPING CHEN, ZHICAI WANG, DAYUN SUI

**Affiliations:** 1 Department of Pharmacology, School of Pharmaceutical Sciences, Jilin University, Changchun, Jilin 130021, P.R. China; 2 Department of Natural Medicinal Chemistry, School of Chemistry, Jilin University, Changchun, Jilin 130021, P.R. China

**Keywords:** *panax quinquefolium* 20(S)-protopanaxadiol saponins, cerebral ischemia, antioxidant, Bcl-2

## Abstract

Oxidative stress is significant in the pathogenesis of cerebral ischemia. *Panax quinquefolium* 20(S)-protopanaxadiol saponins (PQDS) have been demonstrated to exhibit a variety of biological effects in the cardiovascular system as a result of their antioxidant properties. However, little is known regarding the effect of PQDS on cerebral ischemia. The purpose of this study was to investigate whether PQDS exhibited protective effects against cerebral ischemia. A model of cerebral ischemia was induced by middle cerebral artery occlusion (MCAO) in Sprague-Dawley rats. Adult male rats were randomly divided into five groups: Sham, MCAO and PQDS treatment groups at doses of 12.5, 25.0 and 50.0 mg/kg. The effects of PQDS on neurological deficits, cerebral infarct area, brain water content, and the malondialdehyde (MDA) and Ca^2+^ levels and Na^+^-K^+^-ATPase and superoxide dismutase (SOD) activities in the brain tissue were analyzed, and the nitric oxide (NO) content and nitric oxide synthase (NOS) activity in the serum were evaluated. Moreover, the expression of Bcl-2 was analyzed using western blotting. Pretreatment with PQDS (25.0 and 50.0 mg/kg) significantly reduced the neurological deficit score, decreased the infarcted area and decreased the brain water content from 83.09 to 80.27% (P<0.05). In addition, PQDS pretreatment decreased the NOS activity and the NO levels in the serum compared with those in the MCAO group. Furthermore, pretreatment with PQDS (25.0 and 50.0 mg/kg) significantly increased the activities of SOD and Na^+^-K^+^-ATPase and decreased the levels of Ca^2+^ and MDA in the brain tissue (P<0.05) compared with those in the MCAO group. Pretreatment with PQDS (25.0 and 50.0 mg/kg) also increased the protein expression level of Bcl-2 compared with that in the MCAO group. The histopathological results demonstrated the protective effect of PQDS on ischemic injury. The results indicated that PQDS has protective effects against ischemic injury in rats. The mechanism may be associated with the inhibition of oxidative stress and apoptosis.

## Introduction

Ischemic stroke is one of the leading causes of disability in adults worldwide, and the poor prognosis for stroke is largely due to a lack of effective therapies (1). Ischemia and hypoxia lead to the release of cytokines, glutamate excitotoxicity, calcium overload, oxidative stress, disordered metabolism, inflammation and apoptosis in nerve cells (2,3). It has been demonstrated that oxidative stress, inflammation and apoptosis are significant in ischemic injury (4). Reactive oxygen species (ROS) induce lipid peroxidation, protein oxidation and DNA damage (5). Nitric oxide (NO) is one of the ROS and is generated from L-arginine by nitric oxide synthase (NOS). Cerebral ischemia induces excessive NO production, which may aggravate cell injury. Liu *et al* investigated antioxidative or antiapoptotic strategies against ischemic injury (6). Although numerous agents have been demonstrated to exhibit neuroprotective effects in animal experiments, the majority of these agents fail to show efficacy in clinical trials. Therefore, the development of novel agents remains an important issue. Natural products, particularly medicinal plants, have been the subjects of significant focus with regard to neuroprotection in ischemia (7).

*Pana quinquefolium*, also known as American ginseng, is a medicinal herb with a long history in China and other countries. *Pana quinquefolium* has been described to possess anti-stress, anti-diabetic and antitumor effects (8–11). The major active components of *Pana quinquefolium* are ginsenosides, which are divided into the protopanaxadriol, protopanaxatriol and oleanolic acid ginsenosides, according to their structure. *Panax quinquefolium* 20(S)-protopanaxadiol saponins (PQDS) are extracts from the stems and leaf of *Pana quinquefolium* L. and contain numerous ginsenosides, including Rd, Rb2, Rb3, Rc, Rg3 and pF11 (12).

Recently, our laboratory demonstrated that PQDS were able to reduce the infarct size in acute myocardial infarction in rats (13). However, the effects of PQDS on cerebral ischemia have not yet been elucidated. On the basis of previous studies, we hypothesized that PQDS may have a protective effect in cerebral ischemia. Therefore, in the present study, a middle cerebral artery occlusion (MCAO) model of cerebral ischemia was used to examine the protective effects of PQDS in cerebral ischemia and to investigate the potential mechanism underlying the effects of PQDS in rats.

## Materials and methods

### Chemicals and reagents

Malondialdehyde (MDA), superoxide dismutase (SOD), NO, NOS, calcium (Ca^2+^) and Na^+^-K^+^-ATP assay kits were purchased from Nanjing Jiancheng Bioengineering Institute (Nanjing, China). Antibodies against Bcl-2 were obtained from Cell Signaling Technology, Inc. (Beverly, MA, USA), while antibodies against β-actin were purchased from Tianjin Jinmai Gene Mapping Technology Co., Ltd. (Tianjin, China). The PQDS were obtained from Dr Yanping Chen and dissolved in physiological saline for use. All other chemicals were analytical reagents.

### Animals

Male Sprague-Dawley rats (weight, 230–280 g) were purchased from the Experimental Animal Center of Jilin University (Changchun, China). All rats were allowed free access to food and water. The experiments were performed in accordance with the Guide for the Care and Use of Laboratory Animals of Jilin University, and approved by the Ethics Committee of Jilin University.

The rats were randomly divided into five groups (12 rats in each group): i) Sham surgery, where physiological saline was administered to the rats by intraperitoneal (i.p.) injection at a dose of 2 ml/kg; ii) model, where physiological saline was administered to the rats by i.p. injection at a dose of 2 ml/kg; iii) PQDS 12.5 mg/kg, where rats were treated with PQDS by i.p. injection at a dose of 12.5 mg/kg; iv) PQDS 25.0 mg/kg, where rats were treated with PQDS by i.p. injection at a dose of 25.0 mg/kg; and v) PQDS 50.0 mg/kg, where rats were treated with PQDS by i.p. injection at a dose of 50.0 mg/kg. The drug or saline treatment was administered once a day for three consecutive days. Thirty minutes following the final treatment, the rats were anesthetized for the induction of cerebral ischemia.

### Surgical procedures

The MCAO was performed with a modification, as described previously (14). Briefly, the rats in the model and PQDS treatment groups were anesthetized with a 10% i.p. injection of 10% choral hydrate (350 mg/kg). The right common carotid artery, external carotid artery and internal carotid were exposed through a neck incision. The external carotid artery was cut and a monofilament nylon suture with a heated-rounded tip was inserted into the external carotid artery and gently advanced into the internal carotid artery, until a slight resistance was felt. The sham-surgery rats underwent the same surgical procedure with the exception of the MCAO ligation. The suture was tightened around the intraluminal filament to prevent bleeding and the suture was left in place until the rats were sacrificed. The body temperature of the rats was monitored with a rectal probe and was maintained at 37±0.5ºC by a heating pad throughout the surgery.

### Score of neurological deficits

Twenty-four hours subsequent to the induction of the ischemia, the neurological deficits in each rat were assigned a score, using a scale as previously described (15): 0, no observable deficit; 1, difficulty in fully extending the contralateral forelimb; 2, unable to extend the contralateral forelimb; 3, mild circling to the contralateral side; 4, severe circling; and 5, falling to the contralateral side.

### Measurement of infarct area

The infarct volume was assessed using the 2,3,5-triphenyltetrazolium chloride (TTC) method, as described previously (16). The rats were sacrificed and the brains were extracted following the measurement of the neurological deficit score. Each brain was cut into five coronal slices with a blade, and the brain slices were stained with 2% TTC solution at 37ºC for 30 min. Following staining, the areas of cerebral infarction were identified using the different color-tones (white for ischemic cerebral tissue and red for non-ischemic cerebral tissue). The infarct size was calculated as a percentage fraction of the ischemic cerebral tissue in the whole brain.

### Measurement of brain water content

The brain water content was measured using the wet-dry method (17). Following the measurement of the neurological deficit score, the rats were anesthetized using chloral hydrate (350 mg/kg, i.p.) and then sacrificed, prior to the rat brains being rapidly extracted. The pons and olfactory bulb were removed and the wet weight of the brains was measured using an electronic balance. Subsequently, the brains were dried for 24 h at 100ºC in order to obtain the dry weight. The brain water content (BW) was calculated as follows: BW = [(wet weight - dry weight) / wet weight] × 100, and used as an index for brain edema.

### Analysis of MDA and Ca^2+^ levels and the activities of SOD and Na^+^-K^+^-ATPase in the brain tissue

Following the collection of the blood samples, the brains were removed, weighed and homogenized in ice-cold phosphate-buffered saline (PBS). The homogenate was centrifuged (2,500 × g, 15 min) and the supernatant was obtained to measure the activities of SOD and Na^+^-K^+^-ATPase and the levels of MDA and Ca^2+^ using assay kits and a spectrophotometer (7202B; Unico (Shanghai) Instrument Co., Ltd., China), in accordance with the kit manufacturer's instructions.

### Biochemical analysis

Having measured the neurological deficit score, the rats were anesthetized with chloral hydrate (350 mg/kg, i.p.). Blood samples were collected from the abdominal artery into non-heparinized tubes, allowed to clot for 2 h at room temperature and then centrifuged at 2,000 × g for 15 min. The NO level and the activity of NOS were measured using diagnostic kits according to the manufacturer's instructions.

### Histopathological examination

Having measured the neurological deficit score, the rats were sacrificed by decapitation. The brains were rapidly removed and placed into 4% paraformaldehyde solution for one day and then embedded in paraffin. Five-micrometer sections were cut and the brain sections were stained with hematoxylin and eosin (H&E). The brain sections were subsequently examined under a microscope (Nikon ECLIPSE 80i; Nikon, Tokyo, Japan) and photomicrographs were taken.

### Western blotting

The expression of Bcl-2 protein in the rats was analyzed using western blotting. Briefly, the brains were dissected and homogenized with a lysis buffer [1× PBS, 1% NP-40, 0.5% sodium deoxycholate, 0.1% sodium dodecyl sulfate (SDS) and phenylmethylsulfonyl fluoride (PMSF)] to extract the cellular proteins, prior to being centrifuged at 1,200 × g for 15 min at 4ºC. The protein concentration was determined using the bicinchoninic acid (BCA) method. Equal quantities of protein were separated using 12% SDS-polyacrylamide gel electrophoresis (PAGE) and transferred onto polyvinylidene difluoride (PVDF) membranes. Subsequent to blocking with 5% non-fat milk in PBS-Tween-20 (PBST) for 1 h, the membranes were incubated overnight at 4ºC with anti-Bcl-2, and anti-β-actin antibodies. The membranes were then incubated with horseradish peroxidase (HRP)-conjugated secondary antibody (Tianjin Jinmai Gene Mapping Technology Co., Ltd.) against rabbit for 1 h at room temperature. Immunoactive bands were visualized with an enhanced chemiluminescence (ECL) detection system using X-ray film. β-actin was used as an internal control.

### Statistical analysis

All data are expressed as the mean ± standard deviation. Statistical significance was determined using one-way analysis of variance (ANOVA) followed by Dunnett's test. In all cases, P<0.05 was considered to indicate a statistically significant difference.

## Results

### Effect of PQDS on neurological deficits

**Figure 1:**
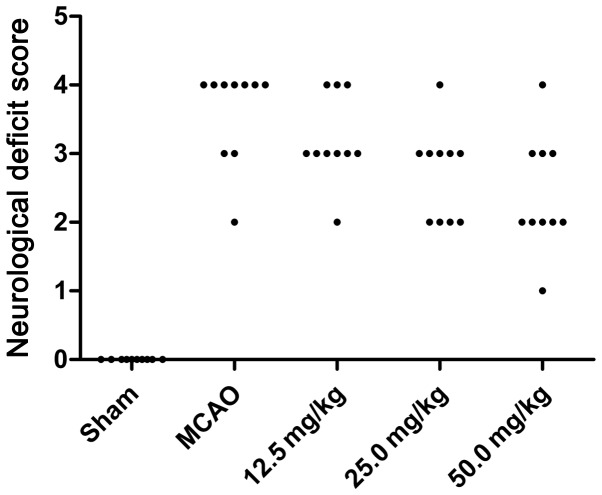
Effect of *Panax quinquefolium* 20(S)-protopanaxadiol saponins (PQDS) on the neurological deficit scores. The neurological deficit scores were significantly increased in the middle cerebral artery occlusion (MCAO) group (P<0.05).

Twenty-four hours subsequent to ischemia, the neurological deficits of the rats were assessed. The neurological deficit scores for the sham, MCAO and PQDS 12.5, 25.0 and 50.0 mg/kg groups were 0, 3.6±0.70, 3.2±0.63, 2.7±0.67 and 2.4±0.84, respectively. The behavioral abnormalities were particularly apparent in the MCAO group, while PQDS treatment (25.0 and 50.0 mg/kg) significantly suppressed the development of the behavioral abnormalities (P<0.05; Table I and Fig. 1).

### Effect of PQDS on brain infarcted area and brain water content

**Figure 2:**
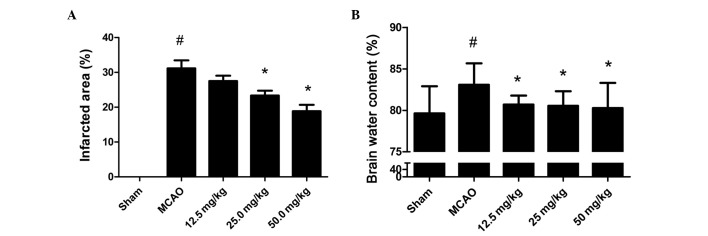
Effect of *Panax quinquefolium* 20(S)-protopanaxadiol saponins (PQDS) on brain infarcted areas and brain water content induced by cerebral ischemia in rats. (A) Infarcted areas and (B) brain water content were measured following 24 h ischemia in Sprague-Dawley rats. Data are presented as the mean ± standard deviation. ^#^P<0.05 compared with sham group; ^*^P<0.05 compared with middle cerebral artery occlusion (MCAO) group.

To investigate the effect of PQDS on cerebral ischemia, the infarcted area and brain water content were measured. As shown in Fig. 2A, no infarcted area was observed in the sham group. The infarcted areas in the PQDS 12.5, 25.0 and 50.0 mg/kg groups were 27.5±3.78, 23.33±3.50 and 18.83±4.49%, respectively, which were lower than that in the MCAO group (31.17±5.56%). The infarcted area tended to be smaller following treatment with 12.5 mg/kg PQDS, although the reductions were not statistically significant.

The brain water content following 24 h ischemia is shown in Fig. 2B. In the sham group, the water content was 79.63±3.29%. Ischemia led to a significant increase in water content in the MCAO group compared with that in the sham group (83.09±2.57%, P<0.05). However, in the PQDS treatment groups, the water content was significantly decreased in a dose-dependent manner. The mean brain water contents were 80.71±1.08, 80.55±1.75 and 80.27±3.03% in the 12.5, 25.0 and 50.0 mg/kg PQDS groups, respectively.

### Effect of PQDS on the levels of MDA and Ca^2+^ and the activities of SOD and Na^+^-K^+^-ATPase in brain tissue

**Figure 3:**
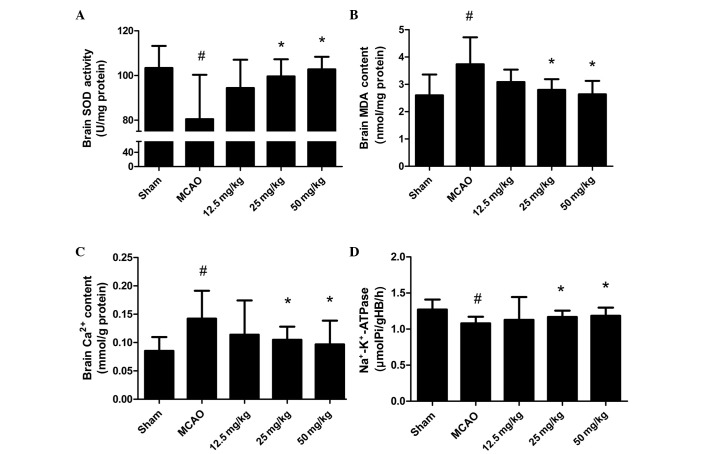
Effect of *Panax quinquefolium* 20(S)-protopanaxadiol saponins (PQDS) on oxidative stress induced by cerebral ischemia in brain tissue. The levels of (A) superoxide dismutase (SOD), (B) malondialdehyde (MDA), (C) Ca^2+^ and (D) Na^+^-K^+^-ATPase were measured respectively following 24 h ischemia. Data are presented as the mean ± standard deviation. ^#^P<0.05 compared with sham group; ^*^P<0.05 compared with middle cerebral artery occlusion (MCAO) group.

To illustrate the effect of PQDS on the oxidative stress induced by MCAO, the levels of MDA and Ca^2+^ and the activities of SOD and Na^+^-K^+^-ATPase were measured. The levels of MDA and Ca^2+^ were increased in the MCAO group compared with those in the sham group (Fig. 3). PQDS treatment (25.0 and 50.0 mg/kg) significantly reduced the levels of MDA and Ca^2+^ in a dose-dependent manner compared with those in the MCAO group. Conversely, significant reductions in the activities of SOD and Na^+^-K^+^-ATPase were observed in the MCAO group, which were significantly attenuated by PQDS treatment (25.0 and 50.0 mg/kg).

### Effect of PQDS on the NO and NOS levels in the serum

**Figure 4:**
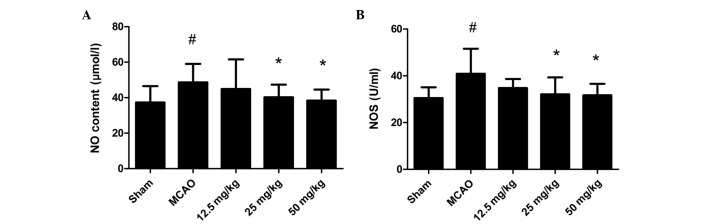
Effect of *Panax quinquefolium* 20(S)-protopanaxadiol saponins (PQDS) on nitric oxide (NO) and nitric oxide synthase (NOS) in the serum. (A) The levels of NO and (B) activity of NOS were measured respectively following 24 h ischemia. Data are presented as the mean ± standard deviation. ^#^P<0.05 compared with sham group, ^*^P<0.05 compared with middle cerebral artery occlusion (MCAO) group.

The changes in the NO and NOS levels are shown in Fig. 4. The NO level and NOS activity were increased in the MCAO group compared with those in the sham group. PQDS treatment significantly reduced the NO level and NOS activity in a dose-dependent manner compared with those in the MCAO group.

### Effect of PQDS on Bcl-2 expression

**Figure 5:**
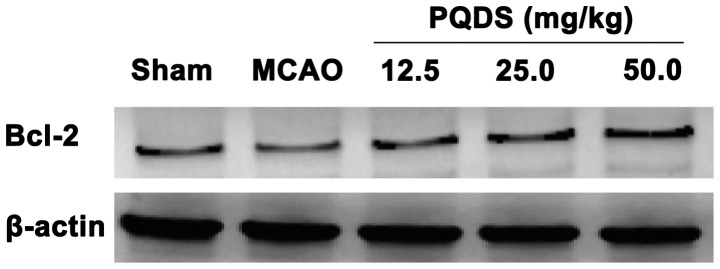
Effect of *Panax quinquefolium* 20(S)-protopanaxadiol saponins (PQDS) on Bcl-2 expression in the brain. The protein expression level of Bcl-2 was assessed using immunoblotting. The level of β-actin was used as the internal control.

To gain an insight into the apoptotic signaling, the expression of the antiapoptotic protein Bcl-2 was analyzed. The level of Bcl-2 protein expressed in the MCAO group was markedly decreased compared with that in the sham group, which was consistent with previous studies (18). Pretreatment with PQDS significantly increased the expression of Bcl-2 compared with that in the MCAO group, suggesting that the protective effects of PQDS may be mediated by the inhibition of apoptosis (Fig. 5).

### Histopathological examination of the brain tissues

**Figure 6:**
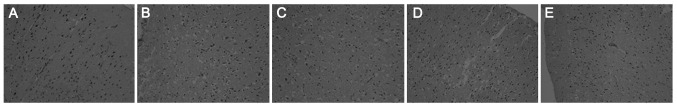
Representative haematoxylin and eosin (H&E) pathological photomicrographs of brain tissue. The brain was excised and fixed with 10% formalin for subsequent H&E staining. The sections were examined under a light microscope, prior to photomicrographs being taken. (A) Sham group; (B) middle cerebral artery occlusion (MCAO) group; (C) *Panax quinquefolium* 20(S)-protopanaxadiol saponins (PQDS) 12.5 mg/kg; (D) PQDS 25.0 mg/kg; and (E) PQDS 50.0 mg/kg. Magnification, ×200.

As shown in Fig. 6, numerous pyramidal neurons were observed in the sham-surgery group, while marked morphological changes were observed in the model group, such as neuronal cell loss, nuclear shrinkage, neuronal vacuolization and dark staining of the neurons. Pretreatment with PQDS (25.0 and 50.0 mg/kg) markedly attenuated these pathological changes; however, 12.5 mg/kg PQDS exhibited no effect.

## Discussion

In our previous study, we demonstrated that PQDS reduced the infarct size in acute myocardial infarction in rats and dogs (13). In the present study, the effects of PQDS on brain damage following MCAO were investigated. The results showed that the MCAO group had significantly decreased neurological function and increased infarct size and brain water content compared with the sham-surgery group. The administration of PQDS effectively reduced the infarct size and brain water content and improved the neurological function and morphological changes in a dose-dependent manner, when administration commenced three days prior to MCAO. These results suggested that PQDS may attenuate cerebral injury in rats. This effect was associated with decreased MDA, NO and Ca^2+^ levels and with increased SOD and Na^+^-K^+^-ATPase activity. It also appeared that PQDS increased the expression of Bcl-2 in cerebral ischemia.

Oxidative stress is significant in ischemic injury. It has been indicated that ROS, such as the superoxide anion and the hydroxyl radical, are produced during ischemia, and that these attack lipids, proteins and DNA in ischemic brain tissue (19). ROS are scavenged by endogenous antioxidant enzymes, such as SOD, catalase (CAT) and glutathione-*S*-transferase (GST), as well as Na^+^-K^+^-ATPase (20). Therefore, measuring the levels of such enzymes enables the amount of oxidative stress to be estimated. The results of the present study showed that cerebral ischemia increased the lipid peroxidation and decreased the activity of endogenous antioxidant enzymes, which was consistent with the results of a previous study (21). Pretreatment with PQDS significantly reduced the level of MDA and increased the activities of SOD and Na^+^-K^+^-ATPase in the brain tissue. These results indicated that the antioxidant properties of PQDS may act as a protective mechanism, by increasing the levels of antioxidant enzymes, such as SOD, to combat the oxidative stress induced by MCAO.

In addition to disordered free radical production and lipid metabolism, Ca^2+^ overload has also been demonstrated to be a risk factor for cerebral ischemic injury (22). During cerebral ischemia, increases in cytoplasmic Ca^2+^ levels are capable of activating phospholipases, endonuclease and proteases, as well as activating enzymes that generate ROS and NO, which participate in cell death (23). Pretreatment with PQDS significantly reduced the level of Ca^2+^ in the brain tissue. This result indicated that the Ca^2+^-lowering property of PQDS might act as a protective mechanism

In addition, the present study demonstrated that the administration of PQDS increased the level of Bcl-2 expression following cerebral ischemia. Oxidative stress, ionic imbalance and excitotoxicity result in nerve cell apoptosis. The Bcl-2 family has been considered to be the most important regulator of apoptosis. The antiapoptotic protein Bcl-2 is capable of preventing cytochrome *c* release into the cytoplasm (24), while the pro-apoptotic protein, Bcl-2-associated X protein (Bax), promotes cell death, unless it is bound by Bcl-2 or Bcl-xL (25). The balance between Bcl-2 and Bax maintains mitochondrial stabilization. The Bcl-2 expression level in the MCAO group was markedly decreased compared with that in the sham group, which is consistent with the results of a previous study (18). Pretreatment with PQDS significantly increased the expression of Bcl-2 compared with that in the MCAO group, suggesting that PQDS may mediate the protective effect against cerebral ischemia by inhibiting apoptosis.

In conclusion, the present study demonstrated the protective effect of PQDS in a rat model of ischemia. The mechanisms were associated with reductions in free radical formation, lipid peroxidation and calcium overload, and an increase in antiapoptotic protein expression. These results suggest that PQDS may have therapeutic potential in the treatment of cerebral ischemic injury. However, further study is required before this is able to be transferred to clinical practice.

## Figures and Tables

**Table I:** Effect of PQDS on neurological deficit scores in rats.

	Score (n)						
		
Group	0	1	2	3	4	5	Average score
Sham	10	-	-	-	-	-	-
MCAO	-	-	1	2	7	-	3.6±0.70^a^
PQDS							
12.5 mg/kg	-	-	1	6	3	-	3.2±0.63
25.0 mg/kg	-	-	4	5	1	-	2.7±0.67^b^
50.0 mg/kg	-	1	5	3	1	-	2.4±0.84^a^

A Mann-Whitney test was used. Each value represents the mean ± standard deviation; n=10 for each group. The neurological deficit scores were significantly increased in the middle cerebral artery occlusion (MCAO) group.

a P<0.05 compared with the sham group,

b P<0.05 compared with the MCAO group. PQDS, *Panax quinquefolium* 20(S)-protopanaxadiol saponins.
